# An Integrated Approach for Delivering Current Astrobiology Research to the General Public

**DOI:** 10.1089/ast.2018.1872

**Published:** 2019-04-25

**Authors:** Erika M. Harnett, Diana Johns, Jennifer Gardner, Kathleen Finneran, Hilarie Davis, Betzalel Massarano

**Affiliations:** ^1^ Astrobiology Program, University of Washington, Seattle, Washington.; ^2^ Pacific Science Center, Seattle, Washington.; ^3^ Technology for Learning Consortium, Inc., Jensen Beach, Florida.; ^4^ Museum of History & Industry, Seattle, Washington.

**Keywords:** Astrobiology education, Biosignatures, Early Earth, Extrasolar planets, Life detection

## Abstract

This article describes a multifaceted approach to delivering results from current research in astrobiology to visitors at Pacific Science Center, along with the evaluated results of the impact of the work. Content was delivered by (1) training scientists to communicate effectively with the public, (2) providing the trained scientists with venues to engage with the public, and (3) creating two Science on Sphere shows that highlight key tenant scientists are investigating, a hands-on activity to facilitate interactive learning, and a temporary exhibit that highlights current research on the topic. Evaluation of visitors who engaged with each element demonstrates that the content had a large impact on both the increase in knowledge of the visitors and the increase of interest in the topic.

## 1. Introduction

When watching the news, it sometimes feels as if wondrous new planets are being discovered everywhere astronomers look in our galaxy, for example: a planet found orbiting Proxima Centauri, the closest star to our solar system, that could have liquid water at its surface (Anglada-Escude *et al.*, 2016); seven Earth-sized planets found orbiting a star 39 light years away, three of which could have liquid water at their surfaces (Gillon *et al.*, 2017); an irregular clump of material orbiting Tabby's star, the shape of which, astronomers have discerned, changes rapidly—what could it be? (Boyajian *et al.*, 2018). The rapid pace of discoveries and tantalizing results that indicate many of these planets are rocky and have the potential for liquid water at their surfaces fuels interest in the quest for finding life somewhere other than Earth.

The news reports of extrasolar planet discoveries are beneficial to the general public in that they keep the public informed and generate excitement regarding science for the next generation. They are also beneficial to the field of astrobiology (AB), as reports of such discoveries cultivate support within the populous for federally funded research in the field. News reports do have the unfortunate trait that they often lack depth of detail due to time or space limitations, and they also do not allow for clarification. When readers or viewers have a question about the report they just saw, how, and if, those inquisitive learners act to learn more is highly dependent upon those resources available to them. The general public, by and large, has little access to scientists who are engaged in current research. This could be due to the fact that the individual lives in an area far from an institute of higher education or due to the low percentage of individuals with STEM degrees in their neighborhood.^1^ The lack of access to expert knowledge may mean that individuals are either limited in their knowledge growth or will rely on sources that are not supported by prevailing research. This lack of access will also disproportionally impact individuals from communities underrepresented in STEM careers and, therefore, become an issue regarding equity.

Informal science education (ISE) institutions, such as science centers and science museums, can offer a point of access for the general public to expert knowledge. ISE institutions can be seen as intended for the general public in that they are less intimidating than college campuses.^2^ ISE institutions can also potentially provide guidance and training for scientists and engineers to enable them to be more effective communicators. This article provides, as a case study, an example of one collaboration between an ISE institution, Pacific Science Center (PSC), and one research program, the Virtual Planetary Laboratory (VPL), that enabled an integrated approach to engaging the general public with results from current research in AB. The article details each manner of facilitating AB content, describes how each pathway connected synergistically with the other pathways, and provides comparative results from summative evaluation.

## 2. Partners

The two institutions involved in the project were PSC, in Seattle, WA, and the VPL, a NASA-funded AB institute, headquartered at the University of Washington, in Seattle, WA. The work was led by the lead author, a VPL scientist, with over a decade of volunteer work at PSC.

PSC began as the United States Science Pavilion, at the 1962 World's Fair. It has since become the home to a vibrant science center that averaged visits by 1,000,000 people each year. This includes typically 60,000 visitors per year who are associated with school groups. PSC has developed multiple ways to engage visitors with current science and engineering developments, with strong ties in research being done at local higher education institutions and research centers.

The VPL has a collection of researchers from 20 different institutions. The VPL research team explores the evolution and limits of planetary habitability by combining laboratory, field, and observational data with state-of-the-art models in radiative transfer, climate, chemistry, geology, astronomy, and biology. By modeling a variety of self-consistent planetary environments, VPL can better determine what it takes to recognize a habitable world, and how we can discriminate between planets with and without life. This is done by conducting research in five interrelated themes as follows:

1. The Earth as an extrasolar planet—How can we characterize the influence of life on Earth and use that information to determine how a distant observer would be capable of detecting the presence of life on Earth? This provides guidance on how we might be able to detect the presence of life on an extrasolar planet.
2. The Earth through time—How we characterize the habitability of Earth is highly dependent on the time in Earth's history we look at. The variety of life on Earth has also varied greatly during Earth's history. This emphasizes how habitability is not a time-static characteristic.
3. The habitable planet—What characteristics, such as location of the planet within its solar system and the location of that solar system within the galaxy, influence how supportive a planet is for the formation and evolution of life? How can we use this to prioritize our search for life, given the menagerie of planets that have already been found?
4. The living planet—How does life interact with its environment, and how does that potentially generate signals (*i.e.*, biosignatures) that could be detected from afar?
5. The observer—How can we improve our ability to find and characterize planets that might be supportive of life?

One of the main goals of the education and public outreach work was to highlight all of these themes and how they all connect into a single picture.

## 3. Content Delivery Pathways

The team employed five different mechanisms to deliver content and engage with the public. These pathways ranged from training scientists to be effective communicators, providing them with venues to communicate their research, and creating permanent and temporary content to be facilitated by both scientists and science center staff.

### 3.1. Science Communication Fellows

The first phase of the project included the initial round of communication training for VPL scientists, who became members of PSC's Science Communication Fellowship program. Fellows undergo training to build skills needed to effectively engage with the public and discuss their research. Fellows-in-training first undergo three 2-h training sessions at PSC, spread over a month and a half. These sessions push the trainees to consider science experiences they found engaging in their youth and what made the experience so engaging. The sessions also inform as to how to effectively communicate topics without the use of jargon and instruct on the role of hands-on experiences in learning. Concurrently, PSC staff work individually with each trainee to develop a hands-on activity that explores some of the fundamental concepts in the trainee's research. The fourth, and final, training session involves a dry-run of trainees facilitating their activities with the teen volunteers in the Discovery Corp program at PSC. Discovery Corp volunteers engage with each trainee, as if a member of the general public, and then provide the trainees with critical feedback on what aspects of their delivery worked well and what did not. This provides the fellows-in-training with the opportunity to revise their activities and delivery before “going live” and interacting with the public.

Upon successful completion of training, Science Communication Fellows then commit to facilitating their activities at a PSC-sponsored event at least three times throughout the year. This typically takes the form of the fellows participating in three of the 3-h “Meet A Scientist” sessions that happen every Saturday afternoon at PSC. During the “Meet A Scientist” sessions, four to six fellows collectively facilitate their activities in a large well-defined space within a main building at PSC. The central location means that the sessions attract not just visitors who are seeking out the engagement, but also the casual visitor. A more detailed description of the entire Science Communication Fellowship program can be found in Selvakumar and Storksdieck (2013). The lead author, and project lead, was part of the first cohort of Science Communication Fellows, detailed in the reference.

During the 5-year project time period, nine AB Science Communication Fellows were trained—two per year in Years 2–4 and three in Year 5. All of the fellows were graduate students within the AB program at the University of Washington, whose studies ranged from topics in space science to extremophiles in Earth's oceans to searching for extrasolar planets. Of the nine AB Science Communication Fellows, three are women and six are men. This is consistent with the demographics of the AB graduate program at the University of Washington. Seven continue to remain active as fellows as of the time of publication of this article. Two became inactive, after multiple years of volunteering as fellows, upon the completion of their PhDs and moving out of the Seattle area.

Ongoing evaluation of the Science Communication Fellowship program as a whole, which included, but was not limited to, AB fellows, has found that a majority of fellows report that participating in the program causes them to think about their research in new and unexpected ways as a direct result of questions fielded from the public. Fellows also overwhelmingly report that the training they received helped them not only feel more comfortable in public speaking, but also more likely to seek out public engagement opportunities. It is also common for fellows to report that the training helps them improve their skills at work, such as mentoring students in a research setting or teaching.

### 3.2. Science in the City

A second component of the content delivery involved PSC's Science in the City program. At the beginning of the project period, the program involved Science Communication Fellows giving short talks about their research at one of three brewpub-type establishments in the greater Seattle area, located 15–20 miles apart to reach geographically diverse audiences. The speaker would give a 20-min talk and then engage in a 40+ min question-and-answer (Q&A) session with the all-ages audience. Owing to changes unconnected with the project, the Science in the City setting was moved to an IMAX theater at PSC in Year 3 of the project. This still enabled the scientist to engage with the public and actually resulted in an increase in the typical audience size (on average ∼20–25 people at the brewpub setting, ∼80–300 people at the PSC setting). This program also connected fellows with a complementary program, called Teen Science Cafes, which occurred at regional teen centers or high schools with an audience entirely composed of high school students.

The Science in the City events, which are scheduled in the early evening, typically attract an older audience than the Meet a Scientist events. The Meet a Scientist events, scheduled during the daytime, typically attract the traditional science center visitors—a family that includes a parent (or parents), preschool to middle school-aged children, and extended members of the family. The Science in the City events also attract families, but typically those with middle to high school-aged children. They also attract adults who do not attend with children.

One specific example of a Science in the City event was a special showing of the 3D IMAX movie “The Search for Life in Space,” which was attended by 185 people.^3^ Before the screening, three members of VPL each presented a short talk about his or her AB research. After the movie, they participated in an audience Q&A session. It was a very enthusiastic crowd with many questions during the official Q&A time, so many that the Q&A session continued outside the venue afterward. Forty-three of the attendees returned paper evaluation forms at the end of the event. A summary of the responses is as follows:

- Participants reported they increased their knowledge of AB (3.97/5.0) in areas such as how exoplanets are identified, the telescopes used, the history of Mars, and all the tools available.
- More than half of the participants plan to find out more, that is, attend other AB talks (67%), attend other Science in the City events (81%), and seek out more information (51%).
- Some of the most interesting things to participants were (i) how interdisciplinary AB is, (ii) how important math is but it is not all math, (iii) the role of computer models in science, (iv) the fact that there is life in extreme conditions even here on Earth, and (v) the methods and computer models used.
- Only 5 of 43 respondents had engaged with other AB content at PSC.
- Attendees heard about the event from Facebook, friends or family, online sites, previous Science in the City events, or UW.
- Most attendees traveled 1–5 miles (43%) or 5–10 miles (31%); fewer traveled 10–15 miles (12%) or >15 miles (14%).

### 3.3. Science on Sphere

Science on Sphere is an education tool developed by NOAA researchers, in which data sets are projected on to a large sphere. These can be static, in time, data sets, such as images of a planet, or time series animations such as the movement of clouds or ocean currents. For the AB project, two shows were developed that highlighted major themes of the VPL project: “Earth—the Pale Blue Dot” (PBD), and “Ancient Earth, Alien Earth” (AE).^4^ The shows were developed to be facilitated by a PSC staff member, who could adapt and adjust some of the script to best match the audience present.

**FIG. 1.**
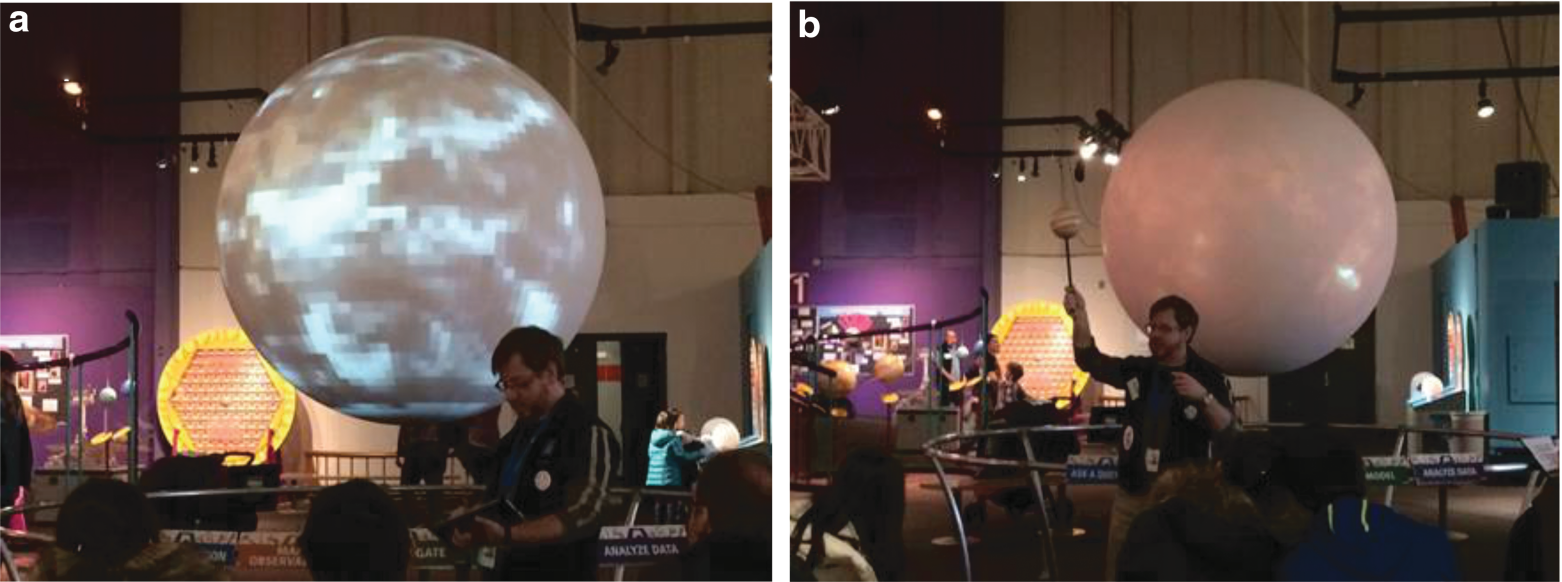
An image of the Earth blurring to a single pixel **(a)** and a PSC interpreter explaining how light from a star dims when a planet passes between it and an observer, with the Sun and Jupiter used as an example **(b)**. PSC, Pacific Science Center.

The PBD show explores the VPL research theme of how might alien observers, using remote sensing techniques, be able to determine that Earth is inhabited by life. Currently, the best astronomers can hope for, when trying to directly observe light from an extrasolar planet, is a single pixel of light, but a vast amount of information can be extracted from that single pixel. Using Earth as an analogue for an extrasolar planet (Fig. 1a), the show demonstrates how reducing the light from Earth to a single pixel would still show a planet that rotates with a 24-h period; has both liquid oceans and landmasses that each reflect different colors of light (more blue light from the oceans and more green or brown from the landmasses), the light reflected from the landmasses changes color on a seasonal timescale due to the growth and die back of vegetation (changing from more green to more brown); and has polar ice caps that grow and recede with the seasons (changes in how all wavelengths of visible light are reflected from the surface). The show also discusses how planets can also be characterized by how they modify the light from their host star, if they transit between us, the observer, and their host star (Fig. 1b).

**FIG. 2.**
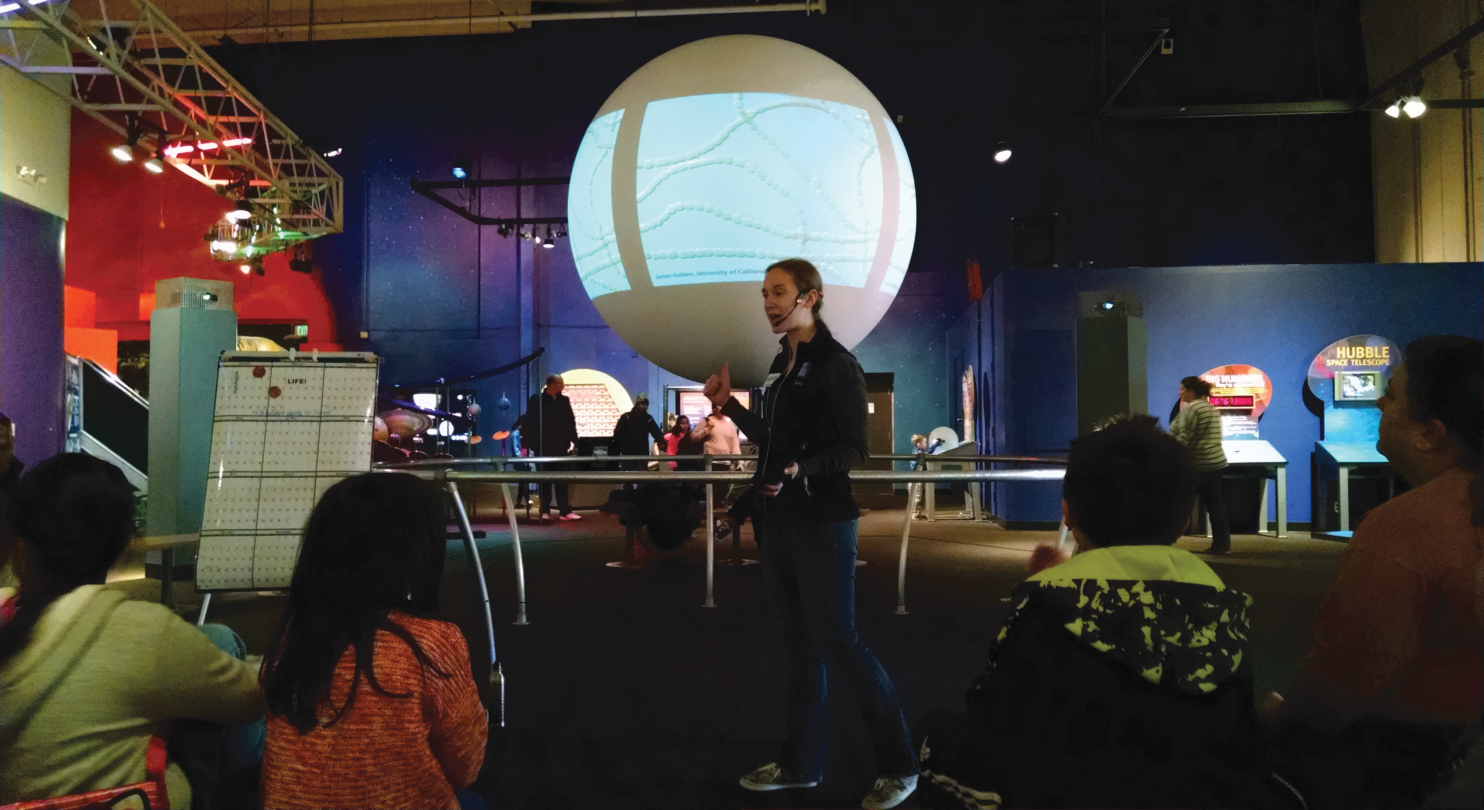
A PSC interpreter discussing the emergence of life on an early Earth.

The AE show explores the VPL research theme of how we characterize the habitability of Earth and how this is greatly dependent upon when, in Earth's history, we look. Early in Earth's history, Earth would have been highly volcanic and hostile to life. As Earth cooled, oceans formed and became the host for life to develop. That life remained ocean bound and only single cellular for almost two billion years (Fig. 2). Life on Earth experienced multiple extinction events, during which large percentages of life died off, but after which the complexity of the life that survived increased. Some extinction events involved impacts from large space objects or wide-spread volcanic eruptions, others appear to have involved how life itself altered the conditions at Earth. The AE show discusses this time line of Earth and how the extent and complexity of life on Earth varied greatly through these different time periods.

Each show took ∼6 months to develop, during which time graphics were either obtained or generated; a show script was generated, piloted, and refined; a training manual of background material was written for show facilitators; and facilitators were trained on presenting a final form of the show script. At each step, feedback was obtained from science experts to ensure accuracy of the science. During the grant period, PBD was presented live 621 times, whereas AE was presented 496 times. The cumulative total of visitors viewing both shows was 10,922.

The summative evaluation of the SOS shows was done through both paper-based questionnaires and observations. For PBD, 55 completed paper surveys were collected (out of 75 total), and for AE, 52 completed paper surveys were collected (out of 67). A total of 61 SOS shows were observed (31 PBD and 30 AE). Of audience members surveyed at the PBD show, 78% self-reported increase in knowledge afterward. Only 62% of audience members surveyed at the AE show reported an increase in knowledge afterward. That visitors indicated less increase in knowledge after viewing the AE show than after viewing the PBD show is interpreted to be related to prior familiarity of the show topics. Some of the topics in the AE show (particularly the history of Earth and the concept of mass extinctions) are topics traditionally covered in the K-12 formal education system. This is supported by the fact that visitors viewing the AE show reported the highest initial self-assessed knowledge of the topic, and the lowest change in knowledge after having viewed the show. Conversely, the topics covered in the PBD show are typically not covered in the formal education system. The viewers of the PBD show reported the lowest initial self-assessed knowledge, but the highest change in self-assessed knowledge, after viewing the show.

### 3.4. Cart activity

**FIG. 3.**
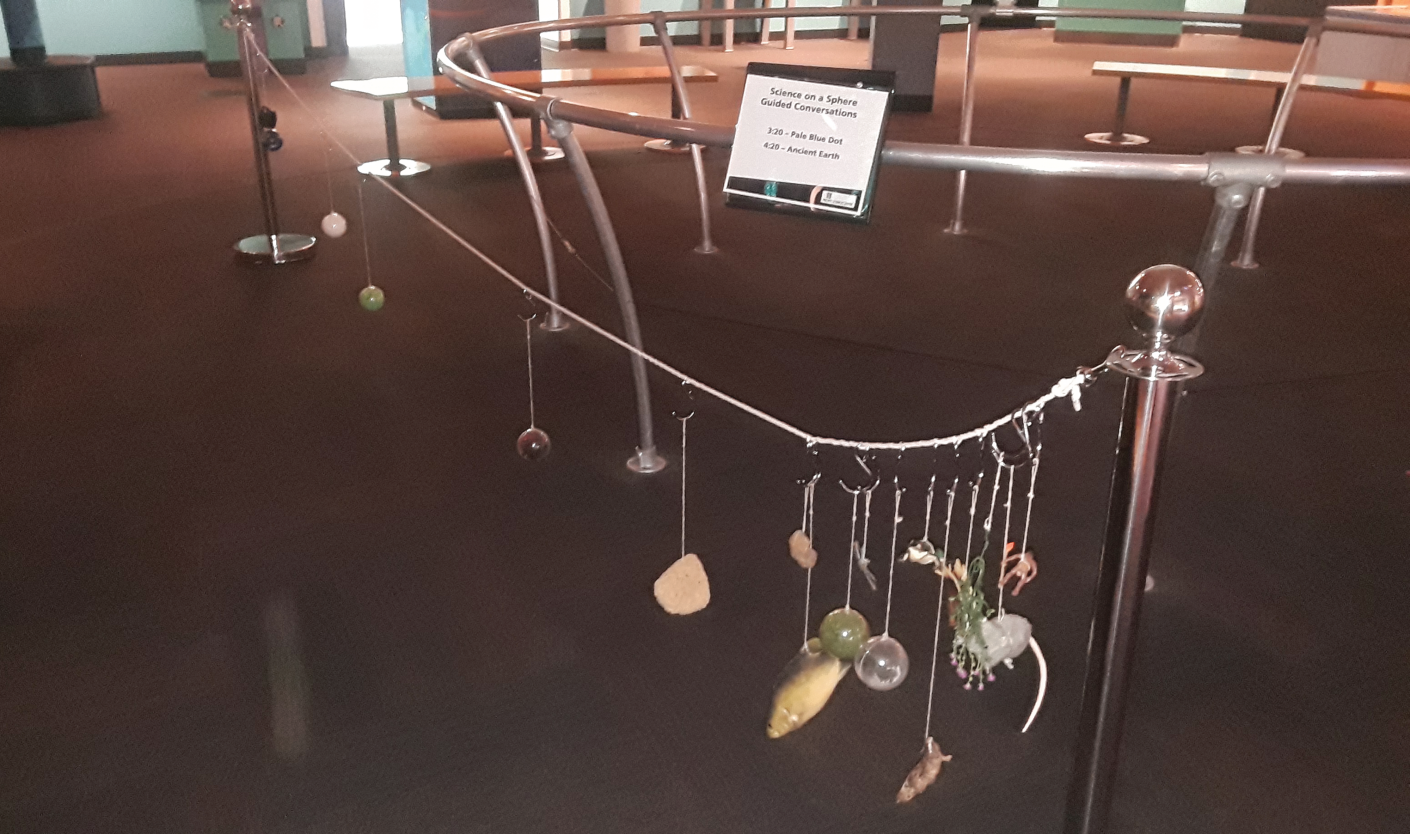
The history of life on Earth cart activity.

A cart activity is temporary activity in which a set of props are used to explore a topic, facilitated by a PSC staff member or volunteer. For the AB project, a cart activity was developed that involves a discussion of the evolution of life on Earth, highlighting elements necessary for life (featured in the exhibit and PBD), as well as the length of time and planetary processes that could be affected by life (AE). Museum visitors (typically younger children) are invited to contemplate when different types of life developed during Earth's history. A rope represents the timeline of Earth, and hooks are placed at intervals representing major evolutionary jumps. Visitors select different types of life from a collection of objects—such as a fuzzy green ball that represented the emergence of cyanobacteria, and a trilobite that represented the Cambrian Explosion. Once the props are identified, guests sort them and arrange them on the timeline in the order of when the events occurred. Educators facilitate the experience by asking questions about what planetary characteristics might have been necessary for these events to occur and how these events might have, in turn, changed those planetary characteristics (Fig. 3). Although designed to be a stand-alone activity, some SOS show facilitators have found it useful to use as part of the AE sphere show, with the cart activity being incorporated into ∼20% of the AE shows.

The cart activity was developed during the final year of the grant period. During a 6-month period, the activity was facilitated with 1397 visitors. (It was staffed for 1 h/day during the work week, and 2 h/day during the weekends.) The activity was evaluated through paper-based questionnaires. Of the 63 questionnaires returned, 47 were completed and analyzed. Average interaction time with the activity was 13 min and 24 s. A total of 79% of respondents self-reported an increase in knowledge afterward.

### 3.5. Portal to Current Research

A capstone to the project included a 6-month exhibit in PSC's Portal to Current Research (P2CR). The P2CR space was created in 2011 with funding from IMLS and NIH. Every 6 months, a new exhibit highlights current research being conducted in the area to broaden the public's awareness of current local research. Exhibit topics have included polar science, chemistry, and neuroscience among others. A similar space, called The Studio, at the PSC, was created in 2012 as part of the Wellbody Academy exhibit and focuses on current health research. It is described in detail in Olson (2015) and Statistical Atlas, Educational Attainment in Seattle, WA.

The exhibit acted as a capstone that connected the other elements of the project together. A topic was selected that was complementary to, but different from, the topic of the Science on Sphere shows. Besides being used with the Science on Sphere shows, the cart activity was frequently set up as a stand-alone activity, near the entrance to the P2CR space, as a way to draw visitors to the P2CR space. In addition, several of the AB fellows facilitated their activity either within or adjacent to the P2CR space, allowing them to also be a live resource for any questions from visitors in the P2CR space. The exhibits were designed so that they could function as both a non-facilitated or facilitated space.

Planning for the AB-themed exhibit began ∼9 months before the exhibit was scheduled to open, with a large brainstorming session in which many VPL team members worked together with PSC staff to define the “big idea” that would tie together all elements in the exhibit. In this case, the “big idea” was defined to be the topic of biosignatures^5^ and the search for life on extrasolar planets. The brainstorming session also highlighted some of the current exciting and cutting-edge research in the topic.

**FIG. 4.**
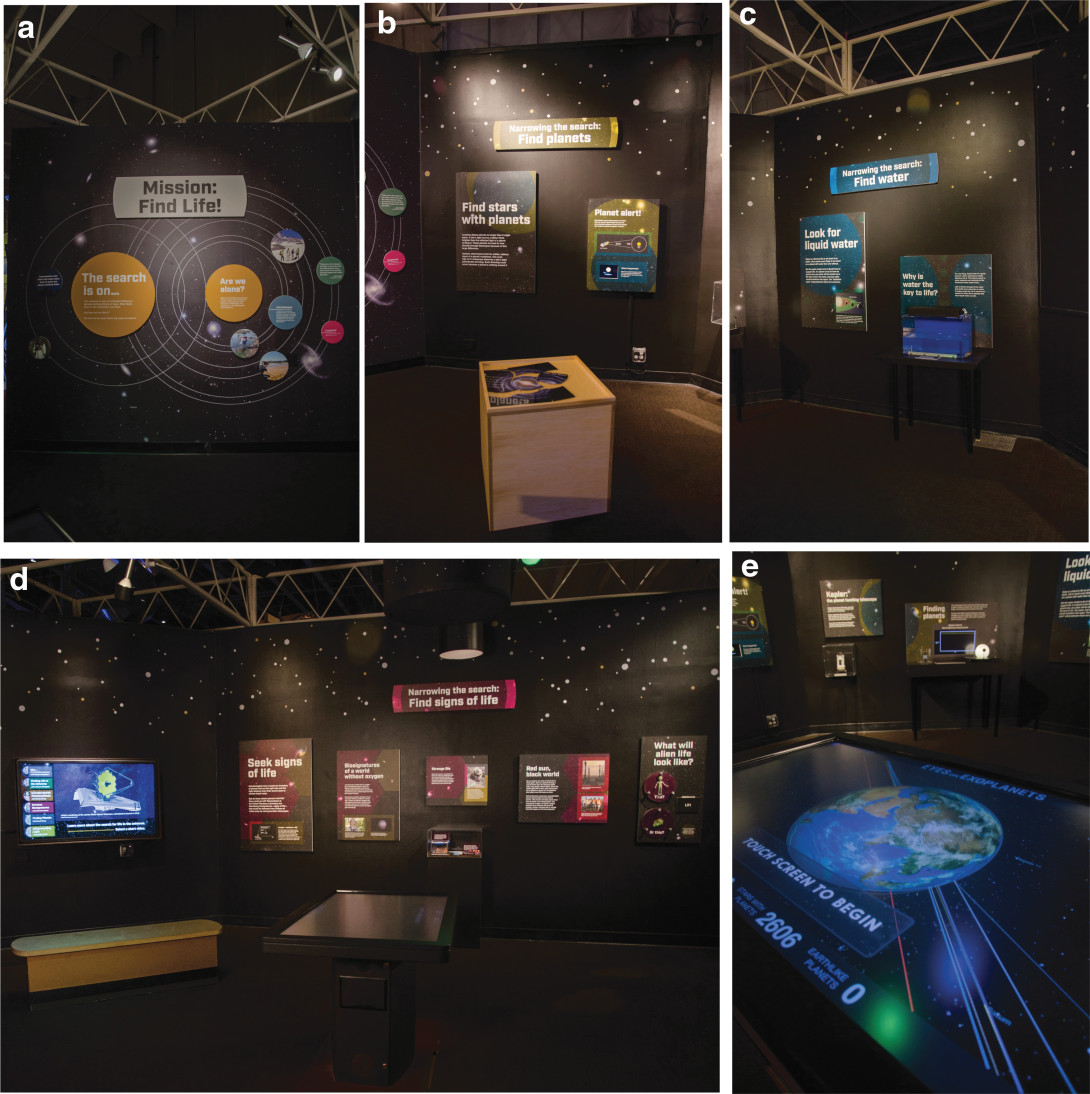
Summary photos of the Mission: Find Life! exhibit. **(a)** The welcoming panel. **(b)** Techniques to find planets. **(c)** The importance of water for the development of life. **(d)** Categorizing the signals of life – Biosignatures. **(e)** The interactive touch table with NASA Eyes on Exoplanets.

**FIG. 5.**
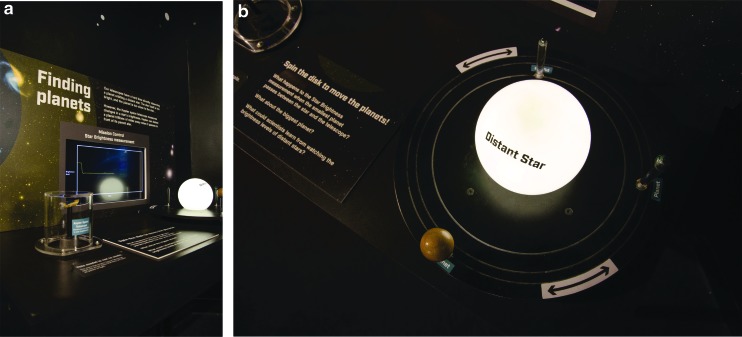
Planet transit activity. **(a)** The full activity, with the light sensor (designed to look like Kepler) on the left, a rotatable dolly with the planets and star on the right, and a screen showing the resulting transit curves in the background. **(b)** The rotatable dolly as viewed from above. Documents to replicate the activity are available as Supplementary Data at https://www.liebertpub.com/suppl/doi/10.1089/ast.2018.1872.

**FIG. 6.**
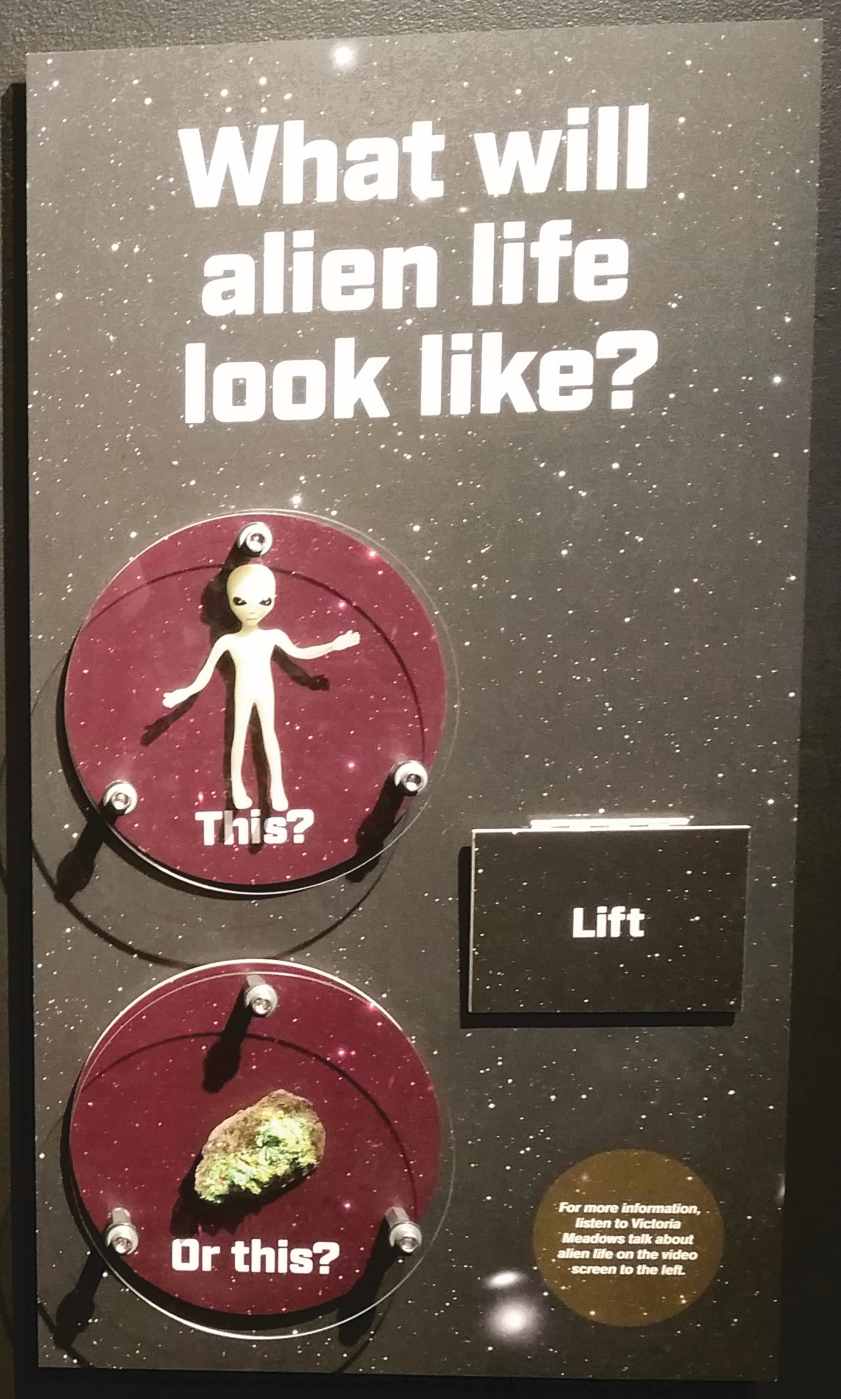
Alien flip board.

The exhibit, titled *Mission: Find Life!*, focused on the big idea that we are narrowing our search for life in the universe to answer the question: Are we alone? Content areas included (1) an introduction that indicated the universe is vast and our need to be smart about the ways in which we search for life, and an overview of VPL's interdisciplinary work (Fig. 4a); (2) narrowing the search: finding stars with planets (Fig. 4b); (3) narrowing the search: finding planets with water (Fig. 4c); and (4) narrowing the search: finding signs of life from a distance (Fig. 4d). Visitors could explore the unusual extrasolar planets on a large touch screen table through the NASA Eyes on Exoplanets program (Fig. 4e). Visitors could also use a model of a distant planetary system to discover how the Kepler space telescope provides scientists with indirect evidence of the existence of distant planets, using a method that observes the dimming of a star when a planet transits between the star and the observer (Fig. 5). Visitors discovered why scientists are specifically looking for planets with liquid water and examined a tank of cyanobacteria. The work of VPL researchers Dr. Niki Parenteau (potential biosignatures from planets with no oxygen) and Dr. Nancy Kiang (potential biosignatures from planets circling a cool M dwarf star) was highlighted, and visitors could examine examples of their field tools and notebooks. Videos were also filmed, featuring VPL researchers^6^ (Prof. Victoria Meadows, Dr. Nancy Kiang, Dr. Niki Parenteau, and Dr. David Crisp) who talked about habitable zones, biosignatures, how to find exoplanets, information carried by light, and their specific research. One activity that was particularly engaging for visitors involved their choosing what life on extrasolar planets is more likely to look like—a little green man or a slime covering a rock (Fig. 6) (the answer is the latter).

Attendance to the exhibit alone was not tracked, but the visitor experience was evaluated. The exhibit was evaluated through both semistructured interviews and timing and tracking observations. A total of 100 interviews were completed and 100 visits were tracked. Of those surveyed, 74% self-reported an increase in knowledge after visiting the exhibit. On average, visitors to the exhibit spent between 2 and 3 min in the exhibit space. A total of 50% of the tracked visitors stopped at the alien flip panel first. This also proved to be the most popular element, with 67% of visitors stopping at that panel (Fig. 6). Of interactive elements, the finding planets carousel (Fig. 5) was the most popular, with 59% of visitors stopping at that element. Exhibit viewers who were interviewed were asked to rate their satisfaction with the exhibit on a scale of 1 to 7 (with 7 being the highest). A total of 92% of interviewees rated the exhibit a 5 or higher, with 28% rating it a 7.

## 4. Benefits and Challenges

A project of this type has many benefits for the partners involved. For the ISE institution, it helps create or solidify a bridge, connecting the ISE with a local university or universities. This relationship can continue beyond the original project period and take on many other facets. The relationship might lead to other funding opportunities of a similar nature. This relationship also creates a natural cohort of content experts who can be relied upon to provide feedback and any future content updates on the part of the ISE institution.

The relationships that developed over time between the museum staff and VPL researchers and AB graduate students were critical to achieve the high quality of the final deliverables. The researchers and graduate students were open and enthusiastic to learning from the PSC staff about effectively interacting with the public, and the PSC staff benefited not only from learning specific content from the experts but also from developing a clearer understanding of the day-to-day work and careers of the scientists. The 5-year project timeline allowed enough time for each deliverable to be developed, reviewed, and revised, which resulted in rich meaningful experiences for museum visitors. The long timeline also presented a small challenge as key PSC staff changed over time.

For the scientists, participation in a Science Communication Fellowship program provides the public with an approachable way to engage with scientists and ask questions that they might otherwise not be able to ask. Scientists are increasingly expected to demonstrate the broader impacts, or societal good, of the work they are requesting grant funds to support. Beyond that, most scientists recognize the importance of a scientifically literate society that supports the funding of basic research, and the importance of outreach to underserved communities to improve the health of STEM. Combined evaluation of the public's interest in AB, before and after viewing the SOS shows and the P2CR exhibit, indicated that 65% of respondents increased their excitement or interest in the topic after engaging with the material (44% reported their interest increased somewhat, whereas 21% reported their interest increased a lot). A total of 63% of those surveyed reported that they were likely or very likely (40% likely and 23% very likely) to seek out more information on AB as a result of engaging with the activity. An interested and excited public is a supportive public.

One challenge that remained for the AB Science Communication Fellows was recruitment of VPL faculty—all of the AB science fellows were graduate students when they joined the program. Although there has been successful recruitment to the overarching Science Communication Fellowship (SCF) program at PSC of full-time professionals from local biomedical research institutions, there are few full-time researchers (faculty or staff) from higher education institutions who entered the program as full-time researchers. The main barrier to recruitment of full-time researchers from higher education institutions is inferred to be the lack of institutional recognition for this type of service when being evaluated for promotion. This is supported by evaluation results from those receiving SCF training—typically a majority of fellows who report that they would like to do more outreach cite a lack of support from supervisors in doing outreach and/or supervisors who do not value outreach, as the main impediment. These challenges are not unique to the SCF program, as the typical reward structure in academia does not tend to include outreach activities or does so in a very minimal manner.^10^ One way to advocate for this type of involvement is its potential for growth into funded opportunities. A faculty member's training and initial involvement can enable future collaboration in STEM education opportunities. These, in turn, may allow the faculty members to diversify their research capacity and sources for potential future funding streams. The training can also be helpful for faculty members who are interested in leadership-type positions that require engagement with a wide spectrum of community members and organizations.

**FIG. 7.**
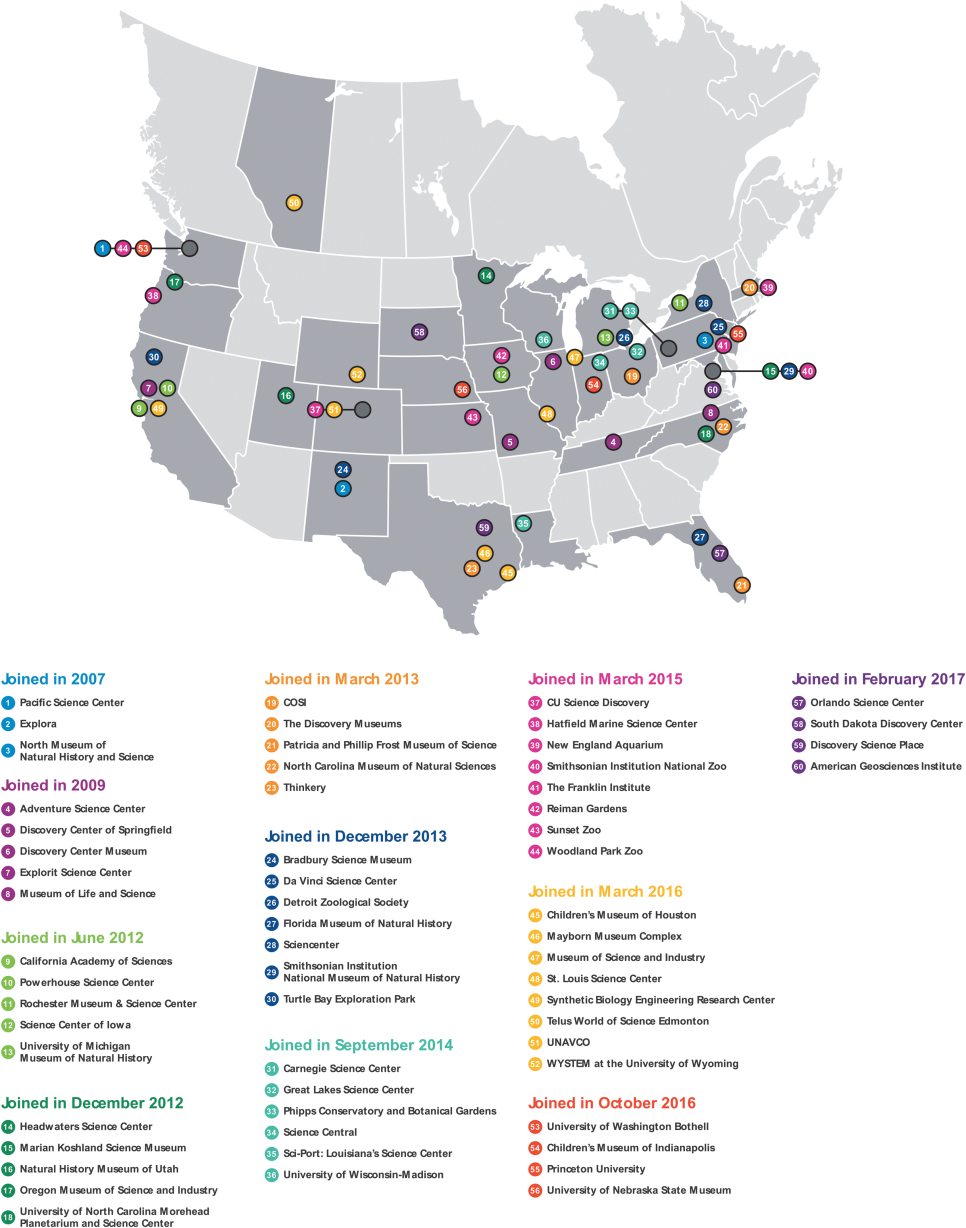
Portal to the public network sites, as of March 2018.

It can be difficult to quantify the benefits from the project, particularly in the case of the science communication training, as the impacts can feed into multiple aspects. For example, in the case of the science communication training, fellows have self-reported improvement in their teaching, as well as improvement in communicating their science to other scientists in a more inclusive and effective manner. Table 1 and Figure 7 detail a semiquantitative cost–benefit analysis of each component for the project. Table 1 shows that the P2CR exhibit and SOS shows were, on average, more successful than previous efforts using these same content delivery methods. Mechanisms were not put in place to assess the experiences of the AB fellows, relative to the experiences of non-AB fellows, as doing so would have removed the anonymity of the AB fellows, due to the small number of AB fellows in each annual cohort of all fellows. The development process of the cart activity was also sufficiently different from previous cart activities such that no comparison could be made with previous activities, from an evaluation standpoint. Both Table 1 and Figure 7 indicate a relative net benefit for all three populations (*i.e.*, scientists, general public, and ISE).

**Table 1.** 

*Activity*	*Goal*	*Measure*	*Result*	*Relative success*
Science on a Sphere	Visitors are increasingly aware of current research and scientists working in this field	Change in visitors' self-reported knowledge of how scientists search for life in the universe	78% of respondents reported an increase in knowledge, on average of 57%	More successful: Previous Science on a Sphere shows resulted in increases in knowledge of 17%–40%
		Change in visitors' self-reported knowledge of the history of life on Earth	62% of respondents reported an increase in knowledge, on average of 48%	
	Visitors become interested and personally engaged in astrobiology and the scientists involved	Change in visitors' self-reported excitement about how scientists search for life in the universe	76% of respondents reported an increase in excitement	As successful: Previous Science on a Sphere shows resulted in 69% of respondents reporting an increase in interest
		Change in visitors' self-reported interest in the history of life on Earth	67% of respondents reported an increase in interest	
Exhibit	Visitors are increasingly aware of current research and scientists working in this field	Change in visitors' self-reported knowledge of how scientists search for life in the universe	74% of respondents reported an increase in knowledge of 54%	More successful: The previous exhibit resulted in 50% of respondents reporting an increase in knowledge of 19%
	Visitors become interested and personally engaged in astrobiology and the scientists involved	Change in visitors' self-reported excitement about how scientists search for life in the universe	51% of respondents reported an increase in excitement	More successful: The previous exhibit resulted in 35% experiencing an increase in appreciation
		Engagement with exhibit	Visitors spent on average between 2 and 3 min on the exhibit	Less successful: In previous exhibits, visitors spent an average of 4–5 min
			92% rated the exhibit 5 or higher out of 7	More successful: In the previous exhibit, 79% rated it 5 or higher
SIP activity	Visitors are increasingly aware of current research and scientists working in this field	Change in visitors' self-reported knowledge of the history of life on Earth	79% of respondents reported an increase in knowledge	No comparison available
	Visitors become interested and personally engaged in astrobiology and the scientists involved	Change in visitors' self-reported interest in the history of life on Earth	81% of respondents reported an increase in interest	
Science Communication Fellows	Fellows increase their understanding of teaching and learning in informal science settings	Fellows' self-reported changes in thinking about teaching and learning	Fellows learned the importance of figuring out what your audience knows first, the value of asking questions, acknowledging that people learn in different ways, that leading people to discover can be more rewarding than explaining, and that it is helpful to boil down concepts to their essence.	
	Fellows increase their comfort and confidence in communicating with public audiences	Change in fellows' self-reported communication skills	All but one respondent felt that their science communication skills improved “some” or “a lot.” 82% report feeling “very equipped” to engage in two-way conversation with visitors	
	Fellows are positively impacted in their interest in a sustained or expanded commitment to participate in public outreach	Fellows' self-reported interest in outreach	All respondents would like to spend “as much as” or “more time” than they currently do engaging with the public about science and research.	

## 5. Tips for Scientists

All of the scientists who participated in the project detailed here found the experience highly rewarding, but becoming involved with, or creating, a program like that described here can initially appear very daunting. This does not need to be. Some tips for scientists considering becoming involved in aspects like those in the project are described hereunder.

Scientists should first discern whether connecting with an ISE institution that is already part of the portal to the public network is possible. The portal to the public network, from which the SCF program arose, was initially three institutions led by the PSC but has since grown to 60 institutions. Figure 7 shows a map of the location of all the ISE institutions that were either part of the original network or have received training from PSC to deploy elements of the portal to the public at their institution. Scientists considering this route on a small scale should plan on writing in funds to pay for the training as part of the broader impacts portion of a grant proposal, or they could inquire whether funds are available through their institution's overhead return to support their training. Cost to train a Science Communication Fellow averages ∼$2500 for the full process.

If access to training through the existing portal to the public network is not available, a scientist's institution may have a communication course available to staff and/or faculty, as part of a University- or College-wide initiative. Many institutions understand the importance of a faculty and staff that are effective communicators with the public. It is worth noting, though, that university-sponsored communication courses can tend to focus on training researchers to communicate effectively with the media, politicians and policymakers and/or the general public at large talks, and these skills can still be useful for learning to avoid jargon. Additional resources may be needed to help the scientist in developing a hands-on activity, through which to engage learners. In many cases, this is a crucial part of the learning experience, especially for children, for whom abstract concepts are difficult to comprehend.

**FIG. 8.**
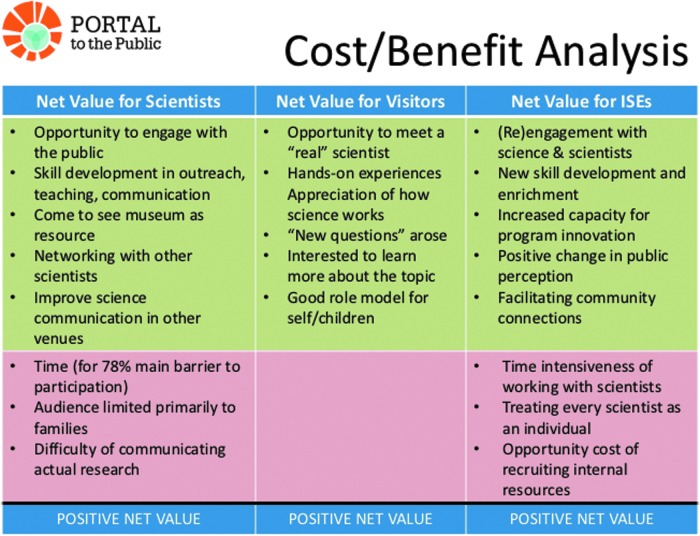
A summary cost–benefit analysis for each group involved in the project.

Scientists should be encouraged and not be intimidated by the thought that they must figure out how to explain their research to every age group. Each person will bring his/her own unique set of knowledge, interests, and context (what is known as constructivist theory [National Research Council, 2000]), and they can help guide the scientist to target the concepts and ideas that the person wishes to learn about. Learning is more than accumulating content knowledge; it is also a social process (National Research Council, 2010). Part of science learning for nonscientists is becoming familiar with the language, tools, and practice of science (National Research Council, 2010). By further sharpening their skills to have two-way meaningful conversations with nonscientists, ranging from adults to young children, scientists can support science learning in informal settings. There is growing evidence that ISE programs can (i) support the development of scientific interests of adults and children, (ii) increase academic achievement for students, and (iii) influence adults' and children's beliefs about their future scientific career possibilities (National Research Council, 2009). In addition, young children are natural and active science learners who, with thoughtful support from adults, will eagerly investigate, experiment with, and learn about the world around them (Nelson *et al*., 2016). A summary of these benefits and their associated costs can be found in Fig. 8.

And finally, when designing hands-on activities, it is critical to think in terms of inclusivity and accessible hands-on activities. If pictures of scientists in action are shown, they should include researchers who represent the full spectrum of our population. Tactile activities that do not require fine motor skills enable full engagement by learners of all abilities and ages. Most importantly, however, scientists involved in these programs and settings should be encouraged to have fun. This will ensure that the public they engage with will sense their enthusiasm, and the end result will be infectious and inspiring to the next generation of scientists.

## Supplementary Material

Supplemental data

## Notes

^1^ As an example, Statistical Atlas, Educational Attainment in Seattle, WA and Statistical Atlas, Occupations in Seattle, WA demonstrate the correlation between neighborhoods, in Seattle, WA, with higher incomes, and neighborhoods with higher percentages of individuals with STEM degrees.

^2^ In some cases, ISE institutions may also offer discounts for lower income families. One example is the Family Access Membership at Pacific Science Center. Membership is $19 for families receiving any form of public assistance.

^3^ PSC has been very successful with the concept of pairing science presentations with movie screenings. A separate project involves scientists giving a short presentation before a screening of a popular science movie at a local theater, with a Q&A session following. These events are 21+ and typically sell out within a day or two of going on sale.

^4^ Show scripts and training manuals can be obtained either from PSC (https://www.pacificsciencecenter.org/materials-and-resources) or the Science on Sphere online repository (https://sos.noaa.gov/Datasets/)

^5^ Biosignatures are characteristics that indicate the presence of life on a planet or moon, and can be sensed remotely. Although there are many ideas, what characteristics actually conclusively indicate the presence of life at a remote location have not yet been identified.

^6^ All of the videos recorded for the P2CR exhibit are available for viewing at “Mission: Find Life!” Video Playlist (2017).

## Abbreviations Used

AE: Ancient Earth, Alien Earth
ISE: Informal science education
P2CR: Portal to Current Research
PDT: “Earth—the Pale Blue Dot”
PSC: Pacific Science Center
SOS: Science on Sphere
UW: University of Washington
VPL: Virtual Planetary Laboratory
