# Supplementary material for: An Integrated Approach for Delivering Current Astrobiology Research to the General Public
Source: Astrobiology. 2019 Apr 25;19(5):696–708. doi: 10.1089/ast.2018.1872 (PMC6486701; doi:10.1089/ast.2018.1872)
Supplement: Supplemental data [file Supp_Data.pdf]

# Supplementary Material

19 May 2017

Astrobiology Transit Interactive Cabinet Build

Operation: User rotates the disk, causing a planet to come between the “star” and the photoresistor mounted inside a small model of the Kepler space telescope. The decrease in incident light is displayed as a falling line graph on the monitor, representing a planetary transit.

Cabinet: The cabinet is constructed of ½” birch plywood joined with wood glue, 18ga brad nails, and 2” corner brackets. The top panel can be removed and the inside accessed by loosening four bolts located on the ends of the cabinet, but the cabinet can be designed to open in any fashion to access the electronics. 3” ventilation holes covered in perforated metal grating were added on both ends and a DC computer case fan installed to provide airflow. It sits on four screw-on IKEA table legs.

Kepler model: The scale model of the Kepler Space Telescope is constructed as follows:

Back end: nylon hex nut, spray-painted grey.

Body: 5/8” OD brass tubing, cut at angle.

Solar panel: card stock, cut/folded to shape and spray-painted metallic blue.

Assembled with superglue and five-minute epoxy.

“Star:” The star consists of a dimmable LED bulb inside a translucent plastic globe diffuser. A bent steel bracket holds the bulb in the center of the globe. The globe is secured to a flanged mount using epoxy. The mount is then screwed to the cabinet top.

“Planets:” These can be any small round object you wish. Drill a ½” hole going about 2/3 of the way through the object, then affix to a ½” OD acrylic or polycarbonate rod using epoxy. For greater resilience, use threaded steel rod instead of acrylic or polycarbonate.

Planetary disk: ½” MDF, cut and rounded on router jig and painted black. Planetary orbit paths drawn on with silver permanent marker. ½” holes drilled for planet rods. Rods can be affixed mechanically (shaft collars, threads, screws, etc.) or with adhesives.

Rotation assembly: This consists of a turntable bearing screwed to the disk and the globe mount. The “star” and globe mount remain stationary while the disk and planets rotate around them.

Astrobiology Transit Parts List

| Item | Part Description | Vendor/Source |
|---|---|---|
| Kepler model | | |
| - Back end | Painted nylon hex nut | any |
| - Body | 5/8” OD brass tube | McMaster-Carr # 8859K37 |
| - Solar panel | Painted card stock | any |
| - Display case | Acrylic box w/ mount flange | Shop-made |
| - Mount | ½” OD steel tube | any |
| Star assembly | | |
| - Light globe | **6” acrylic plastic globe** | **Superior Lighting** |
| - LEDs | **12V 1.2W G4 base LED bulb** | **Amazon** |
| - Dimmer | **PWM dimming controller** | **Amazon** |
| - Power supply | **12VDC 2A wall supply** | **Any** |
| - LED bracket | Bent mild steel, 0.5”x0.125”x7” | Shop-made |
| - Turntable bearing | 6” square lubricated turntable | McMaster-Carr # 1544T1 |
| - Globe mount | 0.5” MDF | Shop-made |
| Electronics | | |
| - Microcontroller | Arduino Uno | Adafruit |
| - Computer | Mac Mini | any |
| - Monitor | 27” LCD monitor | any |
| - HDMI cable | Standard M-M HDMI cable | any |

# Transit Activity

**Rough layout, showing graphic placement, monitor, etc**

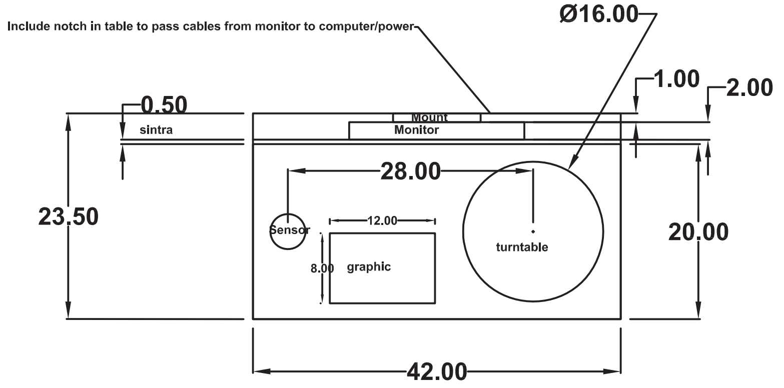


## Top view

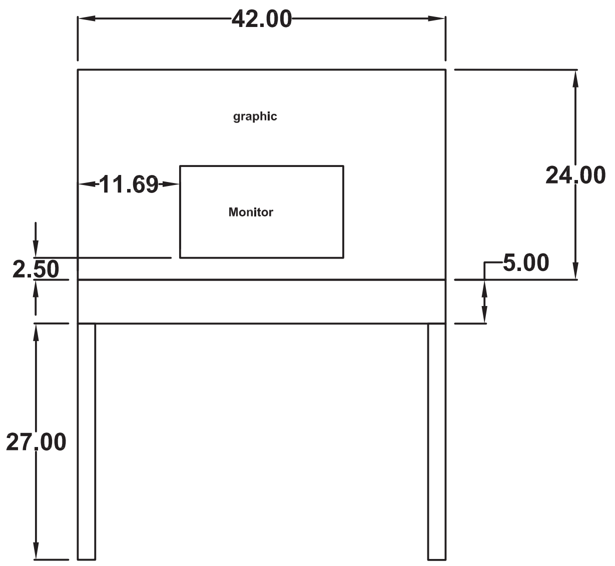


## Front View

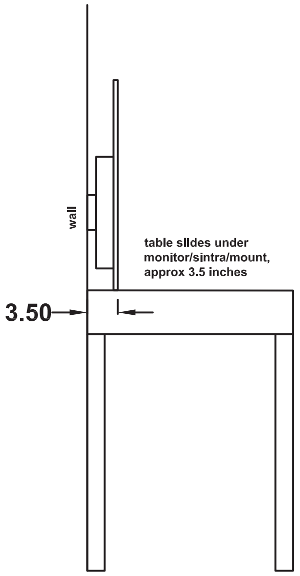


## Side view

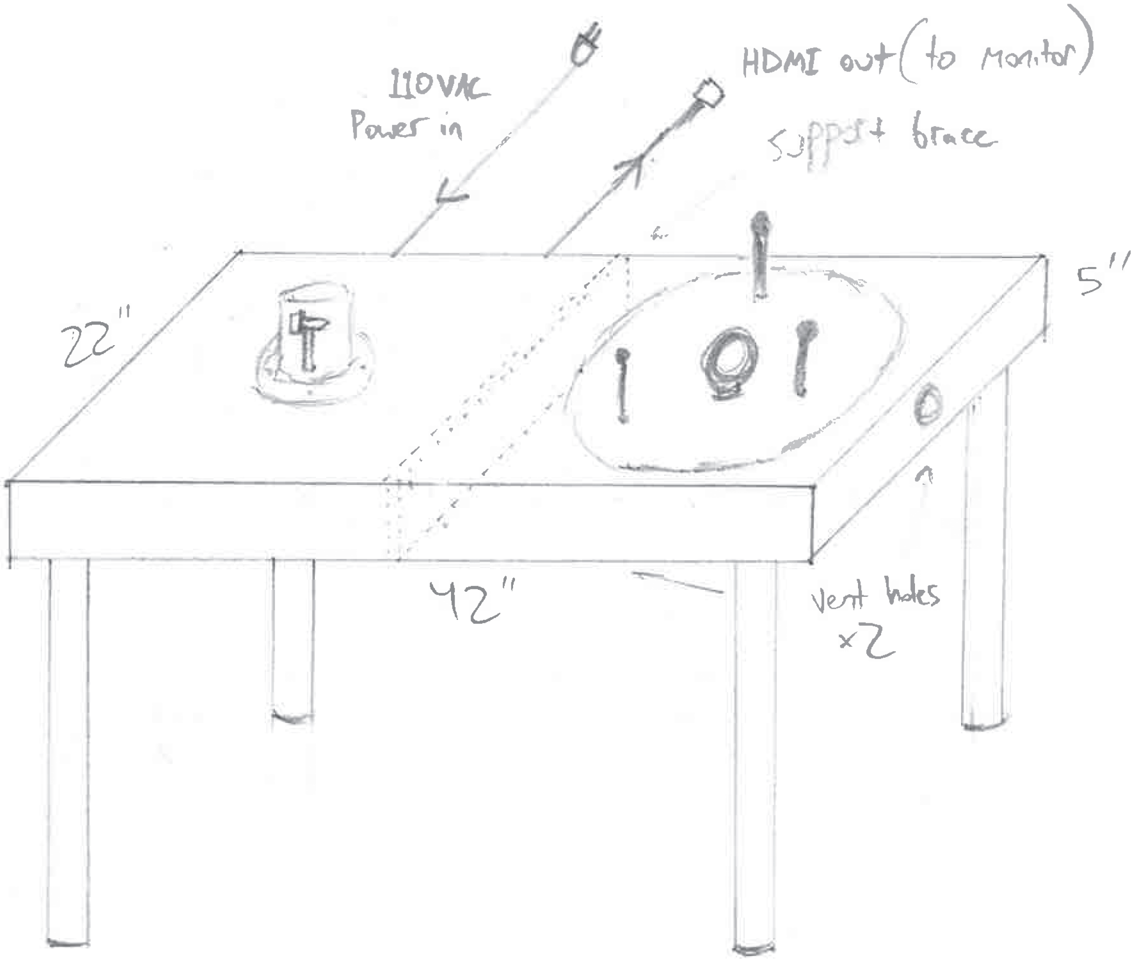


## Star/planet assembly cross-section

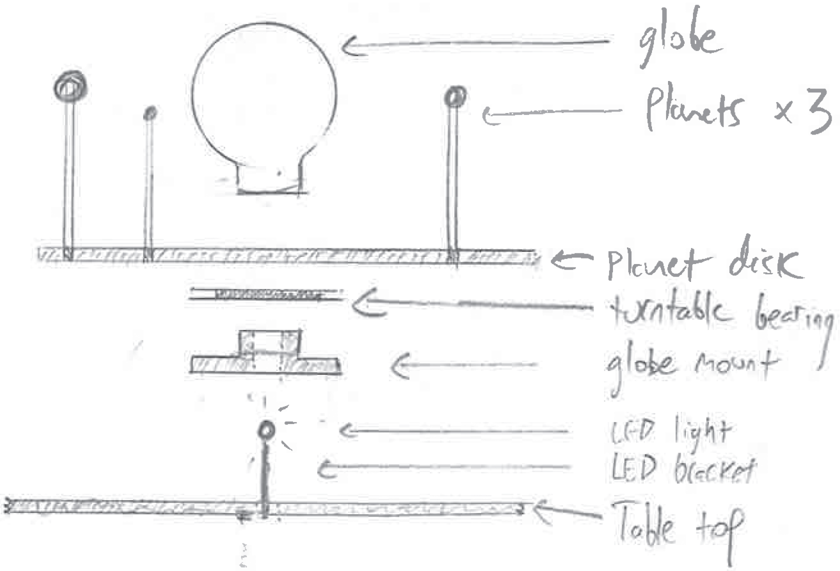

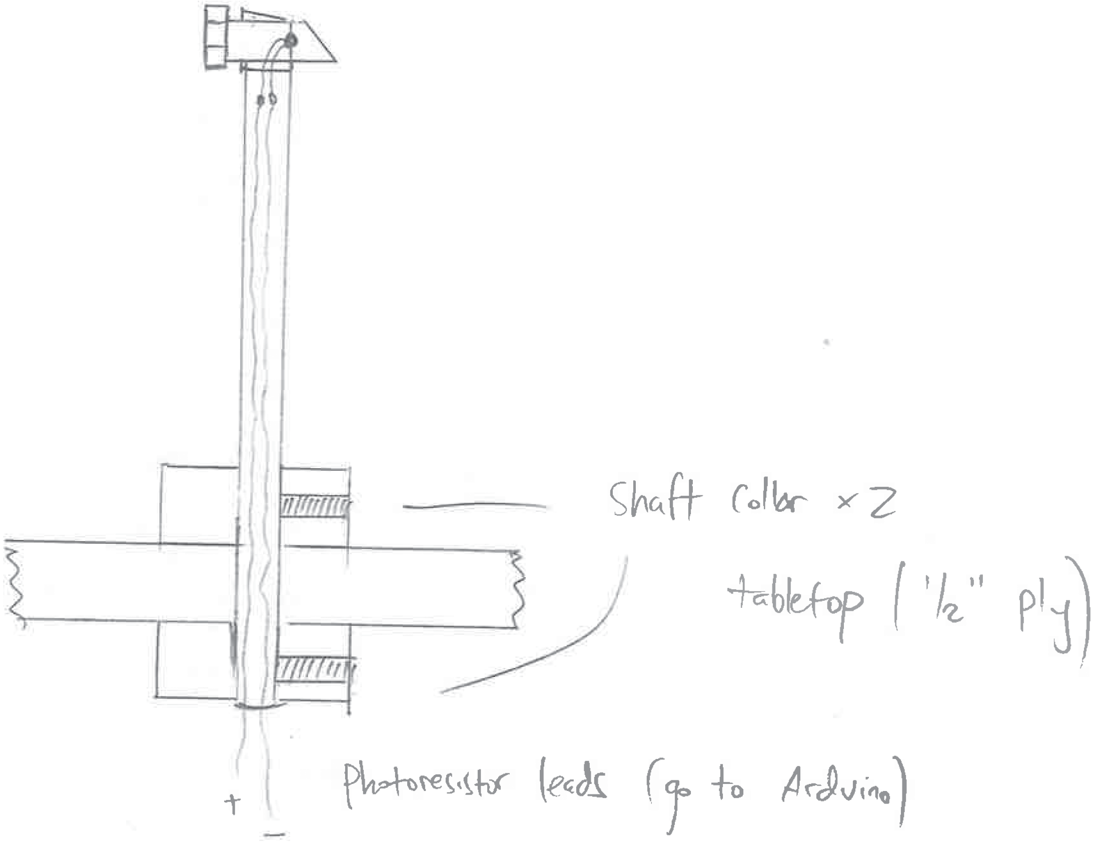
Shaft collar x 2
tablefop (1/2" ply)
Photoresistor leads (go to Arduino)
+
−

Here is the schematic for the Transit exhibit sensor:

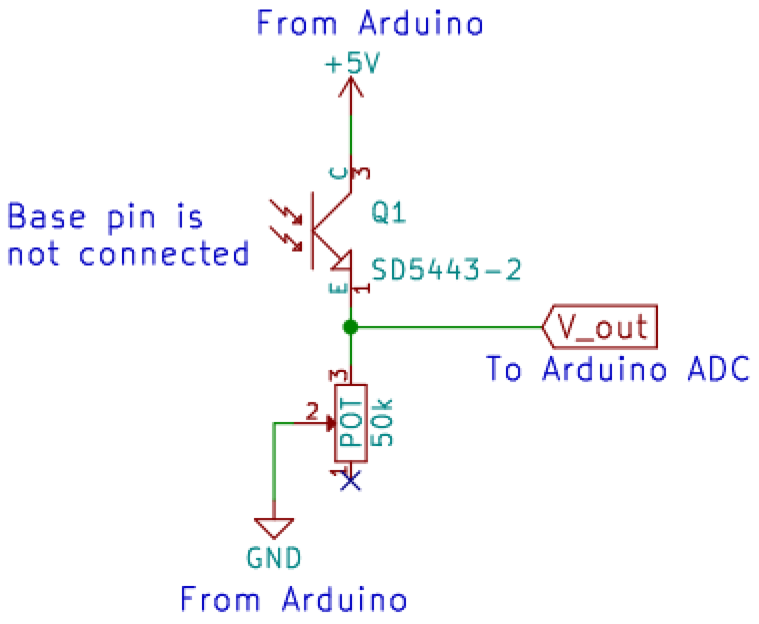


The sensor element is a phototransistor. The SD5443-2 phototransistor was something we had in our parts bins, but many others would work and cost much less. One example is the SFH 313FA from OSRAM. Both of these phototransistors have built-in lenses that accept light from a narrow field of view (18 degrees).

Light hitting the phototransistor causes a current to flow in the phototransistor from the collector through the emitter. That current flows to ground and develops a voltage across the potentiometer (variable resistor). That voltage is connected to an “analog in”, or ADC, pin on an Arduino. Power (5V and ground) comes from an Arduino board as well.

Brighter light intensities cause more current flow, and a higher voltage output. Sensitivity can be adjusted by turning the potentiometer, increasing or decreasing it’s resistance. I did this while observing the running exhibit to get the best results.

Arduino code is used to read the ADC pin and send the data over the USB UART (serial port) to a computer, where it is plotted real-time in a presentable form. I believe our plotting code is written using Processing, which is a good choice.

It is a good idea to shade the sensor element so that ambient light does not get captured (ours is inside the model telescope). Also, the sensor will respond to changes in brightness very quickly, and that includes the rapid effect of light dimmers. Do not use a dimmer on the light source without putting a low pass filter circuit between the phototransistor and the ADC pin. Otherwise, the graph will be very noisy. (If you wanted to be very fancy you could filter digitally in the Arduino code.)

The base pin of the phototransistor is not connected in this circuit, because it is not needed. It could be used to shift the output voltages up, but the same effect could be done in the plotting software more easily.

-Remington Furman, PSC Exhibit Technician, 4-25-2017
